# Cardiovascular abnormalities in chest radiographs of children with pneumonia, Uganda

**DOI:** 10.2471/BLT.22.288801

**Published:** 2023-01-18

**Authors:** Eva Nabawanuka, Faith Ameda, Geoffrey Erem, Samuel Bugeza, RO Opoka, Sarah Kiguli, Denis Amorut, Florence Aloroker, P Olupot-Olupot, Hellen Mnjalla, Ayub Mpoya, Kathryn Maitland

**Affiliations:** a Department of Radiology, School of Medicine, Makerere University, PO Box 7051, Kampala, Uganda.; b Department of Paediatrics, School of Medicine, Makerere University, Kampala, Uganda.; c Mbale Clinical Research Institute, Mbale, Uganda.; d Soroti Regional Referral Hospital, Soroti, Uganda.; e Kenya Medical Research Institute–Wellcome Trust Research Programme, Kilifi, Kenya.; f Department of Infectious Disease and Institute of Global Health and Innovation, Imperial College, London, England.

## Abstract

**Objective:**

To describe chest radiograph findings among children hospitalized with clinically diagnosed severe pneumonia and hypoxaemia at three tertiary facilities in Uganda.

**Methods:**

The study involved clinical and radiograph data on a random sample of 375 children aged 28 days to 12 years enrolled in the Children’s Oxygen Administration Strategies Trial in 2017. Children were hospitalized with a history of respiratory illness and respiratory distress complicated by hypoxaemia, defined as a peripheral oxygen saturation (SpO_2_) < 92%. Radiologists blinded to clinical findings interpreted chest radiographs using standardized World Health Organization method for paediatric chest radiograph reporting. We report clinical and chest radiograph findings using descriptive statistics.

**Findings:**

Overall, 45.9% (172/375) of children had radiological pneumonia, 36.3% (136/375) had a normal chest radiograph and 32.8% (123/375) had other radiograph abnormalities, with or without pneumonia. In addition, 28.3% (106/375) had a cardiovascular abnormality, including 14.9% (56/375) with both pneumonia and another abnormality. There was no significant difference in the prevalence of radiological pneumonia or of cardiovascular abnormalities or in 28-day mortality between children with severe hypoxaemia (SpO_2_: < 80%) and those with mild hypoxaemia (SpO_2_: 80 to < 92%).

**Conclusion:**

Cardiovascular abnormalities were relatively common among children hospitalized with severe pneumonia in Uganda. The standard clinical criteria used to identify pneumonia among children in resource-poor settings were sensitive but lacked specificity. Chest radiographs should be performed routinely for all children with clinical signs of severe pneumonia because it provides useful information on both cardiovascular and respiratory systems.

## Résumé

**Objectif:**

Décrire les résultats des radiographies thoraciques effectuées sur des enfants hospitalisés chez qui une pneumonie aiguë et une hypoxémie ont été diagnostiquées dans trois établissements tertiaires en Ouganda.

**Méthodes:**

L'étude repose sur des données cliniques et radiographiques relatives à un échantillon aléatoire de 375 enfants âgés de 28 jours à 12 ans, recrutés pour l'essai Children's Oxygen Administration Strategies Trial en 2017. Les enfants hospitalisés présentaient des antécédents de maladie et de détresse respiratoires aggravées par une hypoxémie, qui se définit comme une saturation en oxygène périphérique (SpO_2_) < 92%. Sans être informés des résultats cliniques, des radiologues ont interprété les radiographies thoraciques à l'aide de la méthode standardisée de l'Organisation mondiale de la Santé en matière d'établissement de rapports de radiographie thoracique en pédiatrie. Enfin, nous avons consigné les résultats cliniques et radiographiques par le biais de statistiques descriptives.

**Résultats:**

Au total, 45,9% (172/375) des enfants souffraient de pneumonie radiologique, 36,3% (136/375) présentaient une radiographie thoracique normale et 32,8% (123/375) affichaient d'autres anomalies radiographiques, avec ou sans pneumonie. En outre, 28,3% (106/375) avaient développé une anomalie cardiovasculaire; parmi eux, 14,9% (56/375) étaient atteints à la fois d'une pneumonie et d'une autre anomalie. Aucune différence notable n'a été constatée en termes de prévalence pour la pneumonie radiologique, les anomalies cardiovasculaires ou encore la mortalité à 28 jours entre les enfants avec une grave hypoxémie (SpO_2_: < 80%) et ceux avec une légère hypoxémie (SpO_2_: 80 à < 92%).

**Conclusion:**

Les anomalies cardiovasculaires se sont révélées relativement fréquentes parmi les enfants hospitalisés pour une pneumonie sévère en Ouganda. Les critères cliniques standard employés pour identifier une pneumonie chez les enfants issus de milieux où les ressources sont limitées étaient pertinents mais manquaient de précision. Des radiographies thoraciques devraient systématiquement être réalisées sur tous les enfants présentant des signes cliniques de pneumonie sévère car elles fournissent des informations utiles sur les systèmes cardiovasculaire et respiratoire.

## Resumen

**Objetivo:**

Describir los diagnósticos mediante radiografía de tórax en niños hospitalizados con hipoxemia y neumonía grave diagnosticada clínicamente en tres centros de atención especializada de Uganda.

**Métodos:**

El estudio incluyó datos clínicos y radiográficos de una muestra aleatoria de 375 niños de entre 28 días y 12 años incluidos en el ensayo Children's Oxygen Administration Strategies Trial (Ensayo de estrategias de administración de oxígeno en niños) en 2017. Los niños fueron hospitalizados con antecedentes de enfermedad respiratoria y dificultad respiratoria complicada por hipoxemia, definida como una saturación periférica de oxígeno (SpO_2_) <92 %. Los radiólogos no conocían los diagnósticos clínicos e interpretaron las radiografías de tórax utilizando el método estandarizado de la Organización Mundial de la Salud para el informe de radiografías de tórax pediátricas. El presente artículo describe los diagnósticos clínicos y las radiografías de tórax mediante estadísticas descriptivas.

**Resultados:**

En total, el 45,9 % (172/375) de los niños tenían neumonía por radiografía, el 36,3 % (136/375) tenían un tórax normal por radiografía y el 32,8 % (123/375) tenían otras anomalías por radiografía, con o sin neumonía. Además, el 28,3 % (106/375) tenía una anomalía cardiovascular, incluido el 14,9 % (56/375) con neumonía y otra anomalía. No hubo diferencias significativas en la prevalencia de neumonía por radiografía o de anomalías cardiovasculares ni en la mortalidad a los 28 días entre los niños con hipoxemia grave (SpO_2_: <80 %) y los que presentaban hipoxemia leve (SpO_2_: 80 a <92 %).

**Conclusión:**

Las anomalías cardiovasculares fueron relativamente frecuentes entre los niños hospitalizados por neumonía grave en Uganda. Los criterios clínicos estándar utilizados para identificar la neumonía entre los niños que viven en lugares con pocos recursos eran fiables, pero carecían de especificidad. La radiografía de tórax se debería realizar de forma rutinaria en todos los niños con signos clínicos de neumonía grave, ya que proporciona información útil sobre los aparatos cardiovascular y respiratorio.

## ملخص

**الغرض:**

وصف نتائج تصوير الصدر بالأشعة السينية بين الأطفال الذين يخضعون للعلاج بالمستشفى، وتم تشخيص إصابتهم إكلينيكيًا بالتهاب رئوي حاد ونقص تأكسج الدم في ثلاثة مرافق من الدرجة الثالثة في أوغندا.

**الطريقة:**

شملت الدراسة بيانات إكلينيكية ومتعلقة بالأشعة سينية لعينة عشوائية من 375 طفلاً، تتراوح أعمارهم من 28 يومًا إلى 12 عامًا، تم تسجيلهم في تجربة استراتيجيات إدارة الأكسجين للأطفال في عام 2017. خضع للعلاج في المستشفى الأطفال الذين لديهم تاريخ من أمراض الجهاز التنفسي، وضيق في الجهاز التنفسي، وتعرضت للتدهور بسبب نقص تأكسج الدم، وهو ما يُعرَّف بتشبع الأكسجين في الدم المحيطي (SpO2) أقل من %92. قام أخصائيو الأشعة، الذين لم يطلعوا على النتائج الإكلينيكية، بتفسير التصوير بالأشعة السينية للصدر باستخدام الطريقة القياسية لمنظمة الصحة العالمية لإعداد تقارير الأشعة السينية على الصدر للأطفال. نحن نقوم بإعداد تقارير النتائج الإكلينيكية، ونتائج التصوير بالأشعة السينية للصدر باستخدام الإحصاءات الوصفية.

**النتائج:**

بشكل عام، كان %45.9 (172/375) من الأطفال مصابين بالتهاب رئوي إشعاعي، و%36.3 (136/375) لديهم صورة إشعاعية طبيعية للصدر، و%32.8 (123/375) لديهم اعتلالات أخرى في التصوير الإشعاعي، مع أو من دون التهاب رئوي. بالإضافة إلى ذلك، كان %28.3 (106/375) يعانون من اعتلال في القلب والأوعية الدموية، بما في ذلك %14.9 (56/375) مصابين بالتهاب رئوي واعتلال آخر. لم يكن هناك فرق ملحوظ في انتشار الالتهاب الرئوي الإشعاعي، أو اعتلالات القلب والأوعية الدموية، أو في الوفيات في خلال 28 يومًا بين الأطفال المصابين بنقص تأكسج الدم الشديد (SpO2: أقل من %80)، وأولئك المصابين بنقص تأكسج الدم المعتدل (SpO2 : 80 إلى أقل من %92).

**الاستنتاج:**

كانت اعتلالات القلب والأوعية الدموية شائعة نسبيًا بين الأطفال الذين يخضعون للعلاج بالمستشفى للإصابة بالتهاب رئوي شديد في أوغندا. كانت المعايير الإكلينيكية القياسية المستخدمة لاكتشاف الالتهاب الرئوي بين الأطفال في أوضاع فقيرة الموارد حساسة، لكنها تفتقر إلى التخصصية. يجب إجراء تصوير الصدر بالأشعة السينية بشكل روتيني لجميع الأطفال الذين لديهم علامات إكلينيكية للالتهاب الرئوي الحاد لأنها توفر معلومات مفيدة عن كل من القلب والأوعية الدموية والجهاز التنفسي.

## 摘要

**目的:**

旨在描述在乌干达三家三级医院接受住院治疗并经临床诊断患有严重肺炎和低氧血症的儿童的胸部 X 光检测结果。

**方法:**

本研究从参与 2017 年《儿童氧气管理策略试验》的儿童中随机抽样选出了 375 名年龄为出生 28 天至 12 岁的儿童，并纳入了其临床和 X 光检测数据。这些儿童均接受住院治疗，具有呼吸疾病史和低氧血症并发呼吸窘迫，并且经检测，周围氧饱和度 (SpO_2_) < 92%。由事先未看过临床结果的放射科医生采用世界卫生组织的标准化小儿胸部 X 光报告方法解读这些儿童的胸部 X 光检测结果。我们采用描述性统计数据报告临床和胸部 X 光检测结果。

**结果:**

总体而言，45.9% (172/375) 儿童患有放射性肺炎；36.3% (136/375) 儿童的胸部 X 光检测结果正常；32.8% (123/375) 儿童患有其它放射性并发症，不论是否患有肺炎。此外，28.3% (106/375) 儿童患有心血管畸形症，包括 14.9% (56/375) 儿童同时患有肺炎和其他并发症。放射性肺炎、心血管畸形和出生 28 天内死亡的发病率在患有严重低氧血症 (SpO_2_: < 80%) 和患有轻度低氧血症 (SpO_2_: 80 to < 92%) 的儿童之间无明显差异。

**结论:**

在乌干达因严重肺炎接受住院治疗的儿童中，心血管畸形是一种相对常见的疾病。在资源缺乏地区，用于确诊儿童肺炎的标准临床标准比较敏感，但是缺乏具体标准。应定期为出现严重肺炎指征的全部儿童开展胸部 X 光检测，因为该检测可提供关于心血管和呼吸系统的有用信息。

## Резюме

**Цель:**

Описать результаты рентгенографии органов грудной клетки у детей, госпитализированных с клинически диагностированной тяжелой пневмонией и гипоксемией в три специализированных учреждения в Уганде.

**Методы:**

В исследовании использовались клинические данные и данные рентгеновского анализа случайной выборки из 375 детей в возрасте от 28 дней до 12 лет, включенных в исследование Children's Oxygen Administration Strategies Trial в 2017 году. Дети были госпитализированы с респираторным заболеванием и угнетением дыхания в анамнезе, осложненным гипоксемией, определяемой как периферическая кислородная сатурация (SpO2) < 92%. Рентгенологи, не осведомленные о клинических результатах, интерпретировали рентгенограммы органов грудной клетки, используя стандартизированный метод Всемирной организации здравоохранения для проведения рентгенографии органов грудной клетки у детей. О результатах клинических исследований и рентгенографии органов грудной клетки сообщалось с использованием описательной статистики.

**Результаты:**

В общей сложности у 45,9% детей (172/375) были выявлены рентгенологические признаки пневмонии, у 36,3% (136/375) рентгенограмма органов грудной клетки была нормальной, у 32,8% (123/375) на рентгенограммах были выявлены другие отклонения с пневмонией или без нее. Кроме того, у 28,3% пациентов (106/375) наблюдались сердечно-сосудистые аномалии, включая 14,9% пациентов (56/375) с пневмонией и другими аномалиями. Не было выявлено существенной разницы в распространенности рентгенологических признаков пневмонии или сердечно-сосудистых аномалий, а также в показателях смертности в течение 28 дней между детьми с гипоксией тяжелой степени (SpO_2_: < 80%) и детьми с гипоксией легкой степени (SpO_2_: от 80 до < 92%).

**Вывод:**

Сердечно-сосудистые аномалии были относительно распространены среди детей, госпитализированных с тяжелой пневмонией в Уганде. Используемые стандартные клинические критерии для выявления пневмонии у детей в условиях недостатка ресурсов были чувствительными, но не обладали достаточной специфичностью. Всем детям с клиническими признаками тяжелой пневмонии необходимо регулярно проводить рентгенографию органов грудной клетки, поскольку она предоставляет полезную информацию о сердечно-сосудистой и дыхательной системах.

## Introduction

Pneumonia is a leading cause of morbidity and mortality in children globally, accounting for around one million fatalities each year, with the largest burden in low- and middle-income countries.^1^^,^^2^ World Health Organization (WHO) guidelines describe clinical signs and symptoms for diagnosing pneumonia in children.^3^^,^^4^ These guidelines are widely used in Africa, including Uganda. One limitation is that the clinical criteria are broad and are intended to maximize their sensitivity for detecting severe pneumonia at the expense of specificity.^5^ This approach can result in the overdiagnosis of severe pneumonia, thus delaying the definitive diagnosis of other conditions with a similar presentation, potentially leading to mismanagement and increasing the risk of morbidity or mortality.^6^ Although pulse oximetry is helpful for identifying patients with hypoxaemia who require supportive oxygen therapy, its use does not increase the specificity of the guidelines’ diagnostic criteria.^6^

In addition, WHO recommends chest radiography examinations for children with severe pneumonia to confirm the diagnosis and exclude complications. A chest radiograph reporting tool for diagnosing pneumonia in children has been developed to enable researchers to standardize the radiological interpretation of abnormal lung findings.^4^^,^^7^^,^^8^ This WHO-recommended tool classifies significant pathology as the presence of lung consolidation, lung infiltrates or pleural effusion and gives definitions for these entities.^9^ The tool has been evaluated previously and found to be reliable, with good intra- and inter-observer agreement.^8^^–^^10^ Other radiological parameters can also be assessed on chest radiographs, such as the cardiothoracic ratio, which can be used to diagnose cardiomegaly. Findings may also indicate the presence of heart failure, congenital lung or heart abnormalities, or pulmonary oedema, all of which can have a similar clinical presentation to severe pneumonia.

Previously, in the Children’s Oxygen Administration Strategies Trial (COAST) in African children with severe pneumonia,^11^ we used the WHO-recommended tool for interpreting chest radiographs to define radiological pneumonia. The objective of the current study, which was nested within the trial, was to describe chest radiograph findings among Ugandan children hospitalized with a clinical diagnosis of severe pneumonia and hypoxaemia. Better understanding of chest radiograph findings in children who present to hospital with clinical signs of pneumonia can help guide public health measures targeting pneumonia.

## Methods

We performed a cross-sectional study of chest radiograph findings among Ugandan children hospitalized with severe pneumonia and hypoxaemia who were taking part in COAST (ISRCTN15622505; registered: 20 February 2016), which was a multisite, randomized, controlled trial designed to identify the best oxygen delivery strategies for reducing in-hospital morbidity and mortality in African children with respiratory distress complicated by hypoxaemia. The study involved three tertiary health-care facilities in Uganda: (i) Mulago Hospital, which is the national referral hospital for the entire country and a primary health-care facility for the metropolitan area of Kampala, Uganda’s capital;^12^ (ii) the Mbale regional referral hospital, which is a semi-rural government hospital situated 250 km north-east of Kampala; and (iii) the Soroti regional referral hospital, which is a rural government hospital situated 320 km north-east of Kampala. Mulago Hospital has 1790 beds and around 20 000 paediatric admissions annually; Mbale hospital has 470 beds and around 17 000 paediatric admissions annually; and Soroti hospital has 250 beds and around 8000 paediatric admissions annually.^13^

**Fig. 1:**
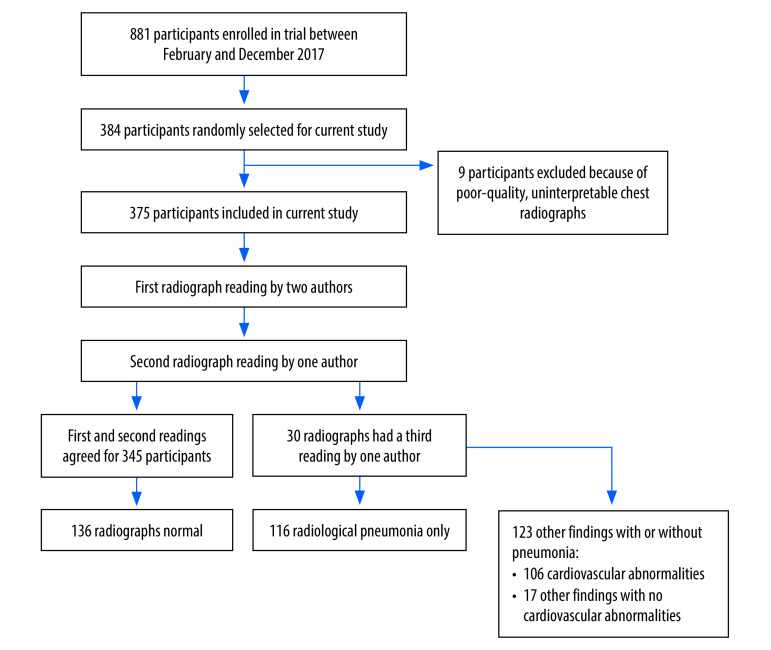
Participant selection for Children’s Oxygen Administration Strategies Trial and radiography findings, chest radiograph study of children hospitalized with severe pneumonia, Uganda, 2017

Note: Children’s Oxygen Administration Strategies Trial.^11^

Children included in the trial were aged between 28 days and 12 years, had a history of respiratory illness and had been hospitalized with respiratory distress complicated by hypoxaemia, which was defined as a peripheral oxygen saturation (SpO_2_) under  92%.^14^ Excluded from the trial were children with chronic lung disease or cyanotic heart disease and those who had received more than 3 hours of oxygen therapy at another centre before referral.^11^ All children were reviewed by a study clinician, and pneumonia was managed in accordance with WHO guidelines.^4^ For this analysis, we used proportionate random sampling to select 384 children who had been enrolled at the three trial sites in 2017 (Fig. 1). The sample size was calculated using the Kish–Leslie formula and was based on the prevalence of radiological pneumonia (as defined by WHO) reported in the Pneumonia Etiology Research for Child Health (PERCH) project, which was performed in a setting similar to ours.^15^^,^^16^ Chest radiographs of the participants selected were retrieved, de-identified and a quality assessment was conducted by one study author. We excluded participants whose radiographs were uninterpretable, such that the presence or absence of radiological pneumonia could not be determined without additional imaging. The final analysis included 375 participants.

A structured, clinical case report form was completed for each child at admission and on reviews at 1, 2, 4, 8, 16, 24, 36 and 48 hours.^11^ Bedside measurements included the respiratory rate, oxygen saturation, anthropometric measurements and chest auscultation; and laboratory investigations included a complete blood count, glucose and lactate point-of-care tests, and blood culture.^14^ Although chest radiograph examinations were ordered at trial admission, they could not be performed immediately in most cases because mobile radiograph facilities were not available. However, as most chest radiographs were conducted before hospital discharge and as the average time to discharge was 4.8 days,^11^ any delay was unlikely to have influenced the appearance of the radiograph because chest radiograph abnormalities have been reported to resolve in around seven days typically.^17^

Anteroposterior views of the chest were obtained at a standard film-focus distance of 100 cm. In addition, posteroanterior views were obtained for older children who were able to stand and obey instructions. All radiographic images were digitized following a standard operating procedure in which a hand-held 20-megapixel camera (Tecno Camon X, Tecno Mobile, Shenzhen, China) was used to take pictures of radiographs mounted on a white-light viewing box. Two radiologists (author EN with either author FA or GE) reviewed the photographic images and reported cases of pneumonia using standardized WHO method for paediatric chest radiograph reporting. If there was a disagreement, a third reader blinded to the initial reports adjudicated. In addition, all radiologists were blinded to the patient’s clinical diagnosis and outcomes and to the trial’s randomization strategy. Radiological pneumonia was diagnosed if the chest radiograph showed evidence of consolidation, infiltrates or pleural effusion. Radiologists also reported clinical findings other than pneumonia. We used a cardiothoracic ratio cut-off of 0.6 to define cardiomegaly. Radiographs showing characteristic chest findings are available in the online repository.^18^

We recorded radiological data, including both pneumonia and non-pneumonia findings, in a database specifically developed for the study using Microsoft Access (Microsoft Corporation, Redmond, United States of America) and later exported to Stata version 13 (StataCorp LLC, College Station, USA) for analysis. Clinical data were obtained from the trial’s Open Clinica database (Open Clinica, Waltham, USA) and also exported to Stata version 13.

### Statistical analysis

We report patients’ characteristics using means and standard deviations for normally distributed variables, and medians and interquartile ranges (IQRs) for skewed variables. Normality was ascertained using the Shapiro–Wilk test and probability plots. We report categorical variables using frequencies and proportions. Patients were divided into two groups according to their peripheral oxygen saturation: (i) those with severe hypoxaemia (i.e. an SpO_2_ under 80%); and (ii) those with mild hypoxaemia (i.e. an SpO_2_ of 80% or above but under 92%). We used frequencies and proportions to describe clinical findings on chest radiographs and to determine their prevalence. The prevalence of radiological pneumonia among study participants was the proportion of all radiographs reviewed that showed evidence of pneumonia, and the prevalence of other chest radiograph findings (e.g. cardiovascular abnormalities) was the proportion of all radiographs reviewed that showed evidence of those findings. The clinical characteristics of participants with severe hypoxaemia and those with mild hypoxaemia were compared using the *χ^2^* test for categorical variables with a cell count of five or over, and using Fischer’s exact test for a cell count under five. Medians were compared using quantile regression. We used a test of proportions, with a 95% confidence interval (95% CI), to test the hypothesis that there was no difference in the proportion of children with specific chest radiograph findings between those with severe and mild hypoxaemia.

Ethical approval for the trial and for this substudy was obtained from the Institutional Research and Ethics Committee of the School of Medicine, Makerere University, Kampala (Ethics reference numbers: 2016–030 and 2019–024, respectively) and from the Research Ethics Committee at Imperial College, London (15IC3100), which was the trial sponsor. Although consent to publish individual data was not obtained from patients or their legal guardians, all data were anonymized before publication.

## Results

Of the 375 participants, 209 (55.7%) were male, 225 (60.0%) were younger than 1 year, 118 (31.5%) were aged between 1 and 5 years and 32 (8.5%) were older than 5 years. Their median age was 9 months (IQR: 4 to 21). The median SpO_2_ on room air at admission was 87% (IQR: 82 to 89). Unsurprisingly, 92.8% (348/375) of children had tachypnoea, 92.5% (347/375) had chest indrawing, 77.1% (289/375) had crepitations and 35.7% (134/375) had an audible wheeze. 

All children were admitted with a working diagnosis of lower respiratory tract infection, although some also had other diagnoses. Overall, 80.3% (301/375) had mild hypoxaemia and 19.7% (74/375) had severe hypoxaemia. In addition, 6.4% (24/374) had severe anaemia (i.e. a haemoglobin concentration less than 5 g/dL), 9.1% (34/372) had a positive malaria rapid diagnostic test result and 3.5% (13/374) had bacteraemia (Table 1). Significantly more children with severe hypoxaemia had clinical cyanosis compared to those with mild hypoxaemia: 9.5% (7/74) versus 1.0% (3/301), respectively (*P* < 0.001). The corresponding proportions in the two groups for other conditions were: (i) 45.9% (34/74) versus 31.9% (96/301), respectively, for clinical shock (*P* < 0.023); (ii) 13.5% (10/74) versus 5.6% (17/301), respectively, for hyperlactataemia (i.e. a blood lactate level > 5 mmol/L) (*P* < 0.019); and (iii) 6.8% (5/74) versus 2.0% (6/301), respectively, for altered consciousness (*P* < 0.03). The median hospital stay was also longer in those with severe hypoxaemia: 5 days (IQR: 4 to 7) versus 4 days (IQR: 3 to 5) in those with mild hypoxaemia (*P* = 0.001). There was no significant difference between these two patient groups in any other baseline clinical parameter.

**Table 1:** Baseline characteristics, chest radiograph study of children hospitalized with severe pneumonia, Uganda, 2017

Parameter at admission	All children (*n* = 375)	Children with mild hypoxaemia^a^ (*n* = 301)	Children with severe hypoxaemia^a^ (*n* = 74)	*P*-value for difference between mild and severe hypoxaemia groups
Age in months, median (IQR)	9 (4 to 21)	9 (4 to 20)	8 (2 to 25)	0.575
Male sex, % (no./n)	55.7 (209/375)	57.4 (173/301)	48.6 (36/74)	0.171
History of fever, % (no./n)	88.8 (333/375)	89.4 (269/301)	86.5 (64/74)	0.481
Respiratory rate in breaths/min, median (IQR)	60 (52 to 68)	58 (52 to 67)	64 (55 to 72)	< 0.001
Tachypnoea, % (no./n)^b^	92.8 (348/375)	91.7 (276/301)	97.3 (72/74)	0.095
Chest indrawing, % (no./n)	92.5 (347/375)	91.4 (275/301)	97.3 (72/74)	0.082
Clinically cyanosed, % (no./n)	2.7 (10/375)	1.0 (3/301)	9.5 (7/74)	< 0.001
Crepitations, % (no./n)^c^	77.3 (289/374)	77.3 (232/300)	77.0 (57/74)	0.955
Wheeze, % (no./n)	35.7 (134/375)	20.9 (63/301)	23.0 (17/74)	0.316
Compensated shock, % (no./n)^d^	34.7 (130/375)	31.9 (96/301)	45.9 (34/74)	0.023
Severe pallor, % (no./n)	8.5 (32/375)	6.3 (19/301)	17.6 (13/74)	0.079
Vomiting or diarrhoea, % (no./n)	29.1 (109/375)	31.2 (94/301)	20.3 (15/74)	0.063
Sunken eyes, % (no./n)	1.9 (7/375)	1.3 (4/301)	4.1 (3/74)	0.121
Reduced skin turgor, % (no./n)	1.9 (7/375)	2.0 (6/301)	1.4 (1/74)	0.715
Altered consciousness, % (no./n)^e^	2.9 (11/375)	2.0 (6/301)	6.8 (5/74)	0.030
Mid upper arm circumference in cm, median (IQR)	14 (13 to 15)	14 (13 to 15)	13.(12 to 15)	0.055
Haemoglobin level in g/dL, median (IQR)	10.3 (8.9 to 11.7)	10.4 (9.1 to 11.6)	9.3 (7.0 to 11.7)	0.001
Severe anaemia, % (no./n)^c,f^	6.4 (24/374)	5.6 (17/301)	9.6 (7/73)	0.079
White blood cell count in cells/µL ( × 10^3^), median (IQR)	11.9 (8.9 to 17.1)	11.9 (9.0 to 16.6)	12.4 (8.3 to 19.5)	0.650
Leucocytosis, % (no./n)^g^	56.8 (213/375)	56.8 (171/301)	56.8 (42/74)	0.993
HIV infection, % (no./n)^c^	1.6 (6/369)	1.0 (3/295)	4.1 (3/74)	0.993
Malaria detected on rapid diagnostic test, % (no./n)^c^	9.1 (34/372)	9.7 (29/299)	6.8 (5/73)	0.717
Malaria detected on microscopy slide, % (no./n)^c^	4.8 (18/373)	4.3 (13/300)	6.8 (5/73)	0.734
Bacteraemia, % (no./n)^c^	3.5 (13/374)	3.0 (9/301)	5.5 (4/73)	0.298
Hypoglycaemia, % (no./n)^h^	2.1 (8/375)	1.7 (5/301)	4.1 (3/74)	0.202
Hyperlactataemia, % (no./n)^i^	7.2 (27/375)	5.6 (17/301)	13.5 (10/74)	0.019
Preadmission antibiotics, % (no./n)^c^	58.8 (218/371)	57.4 (171/298)	64.4 (47/73)	0.128
Hospital stay in days, median (IQR)	4 (3 to 6)	4 (3 to 5)	5 (4 to 7)	0.001
Death within 28 days, % (no./n)^c^	3.0 (11/372)	2.3 (7/299)	5.5 (4/73)	0.156

IQR: interquartile range; HIV: human immunodeficiency virus; SpO_2_: peripheral oxygen saturation.

^a^ We defined mild hypoxaemia as a peripheral oxygen saturation of 80% or above but below 92% and severe hypoxaemia as an SpO_2_ < 80%.

^b^ We defined tachypnoea as a respiratory rate ≥ 60 breaths/min for children aged under 2 months, ≥ 50 breaths/min for those aged over 2 months and under 1 year, ≥ 40 breaths/min for those aged 1 year or more and under 6 years, and ≥ 30 breaths/min for those aged ≥ 6 years.

^c^ Data were either missing, invalid or not recorded for some children.

^d^ We defined compensated shock as one or more signs of impaired perfusion: (i) capillary refill time ≥ 2 s; (ii) a temperature gradient on limbs; (iii) a weak radial pulse; or (iv) severe tachycardia.

^e^ Altered consciousness was determined according to voice and pain responses on the AVPU scale.

^f^ We defined severe anaemia as a haemoglobin concentration < 5 g/dL.

^g^ We defined leucocytosis as a white blood cell count > 11 × 10^3^ cells/µL.

^h^ We defined hypoglycaemia as a blood sugar concentration < 3 mmol/L.

^I^ We defined hyperlactataemia as a blood lactate concentration > 5 mmol/L.

### Radiological pneumonia

Details of the chest radiograph findings are presented in Table 2 and Fig. 1. Overall, 36.3% (136/375) of children had a normal chest radiograph. In addition, 45.9% (172/375) had evidence of pneumonia (i.e. consolidation, other infiltrates, pleural effusion or their combination),^8^ of whom 58.1% (100/172) were younger than 1 year and 54.1% (93/172) were male. Of the 172 children with confirmed pneumonia, 56 (32.6%) had other findings in addition to pneumonia on chest radiograph. Consequently, only 116 of the 375 (30.9%) children had radiological pneumonia as the only finding on chest radiograph. The most common radiological finding indicative of pneumonia was consolidation, which was present in 33.3% (125/372). A lower proportion of children with mild hypoxaemia had consolidation compared with those with severe hypoxaemia: the difference was −14.0 percentage points (95% CI: −26.5 to 15.7). There was no significant difference in the proportion with radiological pneumonia who were male or female (difference: −3.1 percentage points; 95% CI: −13.2 to 7.1) or who were younger than 1 year, or 1 year or older (difference: −1.1 percentage points; 95% CI: −19.2 to 16.9).

**Table 2:** Chest radiograph findings, children hospitalized with severe pneumonia, Uganda, 2017

Chest radiograph finding	No. (%) of children				Difference between mild and severe hypoxaemia groups
	All children (*n* = 375)	Children with mild hypoxaemia^a^ (*n* = 301)	Children with severe hypoxaemia^a^ (*n* = 74)		Percentage points (95% CI)
Normal	136 (36.3)	109 (36.2)	27 (36.5)		−0.3 (−12.5 to 11.9)
**Lung**					
Radiological pneumonia	172 (45.9)	134 (44.5)	38 (51.4)		−6.9 (−19.5 to 5.8)
Consolidation plus either infiltrates or pleural effusion	125 (33.3)	92 (30.6)	33 (44.6)		−14.0 (−26.5 to 15.7)
Other infiltrates only	47 (12.5)	42 (13.9)	5 (6.8)		7.1 (0.3 to 14.1)
Pleural effusion only	0 (0.0)	0 (0.0)	0 (0.0)		NA
**Other findings**					
Cardiovascular abnormalities (with or without pneumonia)	106 (28.3)	88 (29.2)	18 (24.3)		4.9 (−6.1 to 15.9)
Cardiomegaly^b^	44 (11.7)	37 (12.3)	7 (9.5)		2.8 (−22.1 to 27.4)
Cephalization	21 (5.6)	19 (6.3)	2 (2.7)		3.6 (−6.6 to 27.1)
Other cardiovascular findings^c^	41 (10.9)	34 (11.3)	7 (9.5)		1.8 (−13.7 to 28.4)
Parenchymal non-pneumonia findings	4 (1.1)	4 (1.3)	0 (0)		1.3 (−2.4 to 3.7)
Hyperinflation	3 (0.8)	2 (0.7)	1 (1.4)		−0.7 (−11.3 to 5.4)
Cavities	1 (0.3)	1 (0.3)	0 (0)		0.3 (−13.2 to 46.5)
Airway abnormalities, including bronchiolitis	10 (2.7)	9 (2.9)	1 (1.4)		1.5 (−3.3 to 5.2)
Chest wall abnormalities (e.g. spina bifida occulta or scoliosis)	3 (0.8)	2 (0.7)	1 (1.4)		−0.7 (−0.3 to 0.9)

CI: confidence interval; NA: not applicable.

^a^ We defined mild hypoxaemia as a peripheral oxygen saturation (SpO_2_) of 80% or above but  below 92% and severe hypoxaemia as an SpO_2_ < 80%.

^b^ We defined cardiomegaly as a cardiothoracic ratio ≥ 0.6.

^c^ Other cardiovascular findings included vascular pruning, dextrocardia, scimitar syndrome and left atrial enlargement.

Two radiologists differed in their interpretation on 30 of the 375 (8%) chest radiographs and adjudication by another radiologist was required. All disagreements concerned the presence or absence of other infiltrates. There was complete agreement on the presence of consolidation and pleural effusion. Of the 136 children with a normal chest radiograph, 77.2% (105/136) had crackles on clinical examination and 10.3% (14/136) tested positive for malaria, compared with 76.7% (132/172) and 8.7% (15/172), respectively, of those with radiological pneumonia.

### Other chest radiograph findings

Other chest radiograph findings included: (i) cardiovascular abnormalities in 28.3% (106/375); (ii) airway abnormalities in 2.7% (10/375); (iii) parenchymal non-pneumonia abnormalities, such as cavities and hyperinflation, in 1.1% (4/375); and (iv) chest wall abnormalities in 0.8% (3/375). Of the 106 children with cardiovascular abnormalities, 56 (52.8%) were younger than 1 year, 43 (40.6%) were aged between 1 and 5 years, seven (6.6%) were older than 5 years and 63 (59.4%) were male. The most common cardiovascular abnormality was cardiomegaly (41.5%; 44/106). In addition, 19.8% (21/106) of children with cardiovascular abnormalities had cephalization, and 38.7% (41/106) had other cardiovascular findings such as vascular pruning, dextrocardia, scimitar syndrome, left atrial enlargement or simply an abnormal heart shape. Patients were classified on the basis of their key finding, though many had multiple abnormalities.

There was no significant difference between children with mild hypoxaemia and those with severe hypoxaemia in the proportion with cardiovascular abnormalities: the difference was 4.9 percentage points (95% CI: −6.1 to 15.9). Nor did we find a significant difference in the proportion with cardiovascular abnormalities between children who were younger than 1 year, or 1 year or older (difference: 6.9 percentage points; 95% CI: −8.1 to 22.1) or between males and females (difference: 4.2 percentage points; 95% CI: −4.9 to 13.4). Furthermore, there was no significant difference between children with and without cardiovascular abnormalities in any clinical parameter, including 28-day mortality (difference: −8.4 percentage points; 95% CI: −37.2 to 20.4).

## Discussion

We evaluated chest radiograph findings in 375 children aged 28 days to 12 years who were diagnosed with severe pneumonia, the majority (60%) younger than 1 year. We found that almost half had radiological pneumonia, while more than a third had normal chest radiographs. A third of children had other findings, of which more than four fifths had cardiovascular abnormalities. Overall, 56 children had pneumonia with other abnormalities.

The prevalence of radiological pneumonia in these patients was comparable to that in similar studies. For example, a Mozambican study involving children aged 0 to 23 months who were diagnosed with severe pneumonia on the basis of clinical observations and chest radiographs interpreted using the same WHO standardized radiograph reporting format we employed, found the prevalence of radiological pneumonia to be 43%.^19^ Interestingly, the Mozambican study was conducted before vaccination with the decavalent pneumococcal conjugate vaccine (PCV10) was introduced, whereas our study was conducted when national coverage was 64% for the trivalent pneumococcal conjugate vaccine and 79% for the combined vaccine against diphtheria, tetanus, pertussis, hepatitis B, polio and *Haemophilus influenzae* type b (DTaP-HepB-IPV-Hib).^20^ As the vaccination status of trial participants was not recorded, we could not link this data to radiography findings. A second study, the multinational PERCH project, found a prevalence of radiological pneumonia of 54% (range across sites: 35 to 64%) among children aged 1 to 59 months hospitalized with WHO-defined severe or very severe pneumonia (but not necessarily with hypoxaemia) in countries where vaccination with PCV10 and *H. influenzae* type b vaccine had largely been introduced.^15^ Neither of these two studies reported cardiovascular or cardiac anomalies. 

Our study considered hypoxaemia in addition to severe pneumonia, but found no significant difference in the proportion of children with radiological pneumonia between those with severe or mild hypoxaemia. Our finding that more than one third of children had normal chest radiographs was similar to that of an Ethiopian study of 122 children aged 3 months to 14 years who were hospitalized with a diagnosis of severe pneumonia, which reported 51.6% with no radiological evidence of pneumonia.^21^ In the COAST study, we reported a low level of bacteraemia,^11^ which suggests that many of our patients may have had viral pneumonia with no specific changes on chest radiograph.^22^ In agreement with other authors,^23^^–^^25^ we found no correlation between radiological pneumonia and the presence of crepitations on chest auscultation.

Two advantages of our study were that radiographs were interpreted by trained radiologists and that their reports were comprehensive and included cardiovascular abnormalities. The most common cardiovascular abnormality was cardiomegaly. Although there is a dearth of data on the prevalence of cardiovascular abnormalities in Ugandan children, researchers estimated that around 8300 Ugandan children are born annually with congenital heart disease, which corresponds to a rate of 36.6 per thousand live births annually.^26^ Although chest radiograph is not the gold standard for diagnosing cardiovascular disease, it provides preliminary diagnostic information that can identify individuals for referral to echocardiography and other specific tests.^27^^–^^29^ In 2001, researchers evaluated chest radiograph and echocardiography data from 95 children aged between 2 days and 19 years who attended an outpatient clinic and found that the cardiothoracic ratio was an acceptable predictor of cardiac enlargement.^30^ A cardiothoracic ratio of 0.6 had a specificity of 93.4% for identifying cardiomegaly against the gold standard of echocardiography. The 44 children found to have a cardiothoracic ratio of 0.6 or greater in our study had not previously been diagnosed with a cardiac condition (in fact, it was an exclusion criterion) even though the prevalence of paediatric cardiac disease is relatively high in the study setting.^26^ If a chest radiograph had not been performed to confirm the clinical diagnosis of pneumonia in these children, diagnosis and treatment of the heart condition could have been delayed.

The relatively high proportion of abnormal cardiovascular findings among children with severe pneumonia in our study may have occurred because cardiovascular disease is a risk factor for pneumonia.^31^ However, another possible explanation is that children with a cardiovascular illness may present with acute breathing difficulties, cyanosis, lethargy or another danger sign specified by the guidelines for diagnosing severe pneumonia.^4^

Our study confirms that the specificity of the clinical criteria used to diagnose pneumonia in children, especially in low-resource settings, is poor.^5^^,^^32^ In Africa, the specificity of these criteria is further weakened in areas where malaria is endemic because the clinical complications of severe malaria include respiratory distress (i.e. Kussmaul breathing).^33^^,^^34^ However, relatively few children in our study had a diagnosis of malaria.

One limitation of our study was the lack of blood gas analysis (to provide evidence of hypercarbia) and of other investigations (e.g. echocardiography and molecular bacterial and viral analysis) that could be used to correlate clinical and radiological findings. Another limitation is that the statistical accuracy of chest radiography for identifying pneumonia is highly variable.^35^ We tried to minimize inaccuracies by ensuring that radiological pneumonia was identified on radiographs independently by more than one trained radiologist using a standardized WHO tool. Although we failed to find a significant association between hypoxaemia severity and any clinical finding, the 95% CIs were wide, which suggests that a larger sample may be needed to estimate differences with good precision. Nevertheless, we were able to report other abnormal findings, such as cardiovascular conditions, which could be important for children with putative pneumonia.

In conclusion, we found that the clinical criteria used to identify pneumonia among children in resource-poor settings were sensitive but lacked specificity. In our study, a substantial proportion of children had radiological evidence of cardiovascular abnormalities that required further investigation, which is particularly important for children with unresolved hypoxaemia. Consequently, we recommend that chest radiographs should be performed routinely for all children with clinical signs of severe pneumonia because it simultaneously provides information on both cardiovascular and respiratory systems that can be used to improve patient management.
